# High Prevalence of Asymptomatic Sexually Transmitted Infections among Men Who Have Sex with Men

**DOI:** 10.3390/jcm3041386

**Published:** 2014-12-12

**Authors:** Patrick Philibert, Hacène Khiri, Guillaume Pénaranda, Claire Camus, Marie-Pierre Drogoul, Philippe Halfon

**Affiliations:** 1 Département de Médecine Interne et Maladies Infectieuses, Hôpital Européen, 13003 Marseille, France; E-Mails: pp.doc@wanadoo.fr (P.P.); mp_drogoul@CH-AmbroisePare.fr (M.-P.D.); 2 Département de Recherche et Développement, Laboratoire Alphabio, 1 rue Melchior Guinot, 13003 Marseille, France; E-Mails: h.khiri@alphabio.fr (H.K.); g.penaranda@alphabio.fr (G.P.); c.camus@alphabio.fr (C.C.)

**Keywords:** sexually transmitted infection, men who have sex with men, high risk HPV infection, neisseria gonorrhoeae, chlamydia trachomatis, mycoplasma genitalium

## Abstract

**Background:** Men who have sex with men (MSM) are disproportionately affected by sexually transmitted infection. The aim of this cross-sectional study is to prospectively detect the prevalence of chlamydia trachomatis (CT), neisseria gonorrhoeae (NG), mycoplasma genitalium (MG), and high risk human papillomavirus (HR-HPV), and syphilis in a population of asymptomatic sexually active MSM. **Methods:** Rectal, pharyngeal, and urine samples for CT, NG, MG, and HR-HPV were analyzed in 116 MSM patients attending the clinic for their routine follow-up during the period the study was conducted: 99 patients were issued from the clinic routine follow-up for their HIV infection, and 17 attended the clinic because they were sexual partners of an HIV infected male. **Results:** An STI was found in 16% of the patients (19/116), with at least one bacterial strain (CT, NG, or MG) found in one site (the pharynx, rectum, or urine). **Conclusions:** In this study, 16% of the MSM reporting recent RAI were asymptomatic carriers of rectal CT, NG, or MG. According to the high prevalence of asymptomatic STIs found in our MSM population and in other studies, prevention efforts in the form of counseling about the risk of STI need to be done in the population of MSM.

## 1. Introduction

Men who have sex with men (MSM) are disproportionately affected by sexually transmitted infection (STI) and HIV; outbreaks and increasing trends have been recently reported [1,2,3]. STI may increase the risk of contracting HIV through receptive anal intercourse (RAI), and insertive anal intercourse [1,2]. Pharyngeal and rectal infections in sexually active MSM could remain undetected and thus transmissible if screening is not routinely offered [2]. There is a particular urgency to reduce new infections because multiple drug resistant strains of gonorrhoea have been isolated more frequently. The anatomic distribution of chlamydia trachomatis (CT), neisseria gonorrhoeae (NG), mycoplasma genitalium (MG), and high risk human papillomavirus (HR-HPV) infections in MSM, and the prevalence of these infections have been rarely characterized in asymptomatic MSM in France. The aim of this cross-sectional study is to prospectively detect the prevalence of CT, NG, MG, HR-HPV, and syphilis in a population of asymptomatic sexually active MSM.

## 2. Material and Methods

Patients were consecutively included from August 2012 to December 2012 at an urban public clinic for sexually transmitted diseases in Marseille (France). Approximately 400 MSM benefit from a routine follow-up in the clinic. For this study, rectal, pharyngeal, and urine samples for CT, NG, MG, and HR-HPV were analyzed in 116 MSM patients attending the clinic for their routine follow-up during the period the study was conducted: 99 patients were issued from the clinic routine follow-up for their HIV infection, and 17 attended the clinic because they were unprotected sexual partners of an HIV infected male. MSM was officially disclosed by the patients during a follow-up visit with the practitioner. All patients were tested at all anatomic sites (rectal, oropharyngeal, urethral) regardless of symptoms and reported sites of exposure. All patients reported recent sexual intercourse. Patients were predominantly Caucasian (96%), with a mean age of 46.4 ± 9.4; 99 patients were HIV positive and 17 were HIV negative. CT, NG, MG and HR-HPV were assessed using NAT Cobas Taqman 4800 (Roche Diagnostics France, Meylan, France), and syphilis antibodies (TPHA and VDRL) were screened in these patients [4].

As this non interventional study included only patients issued from routine follow up, declaration to ethic committee is not mandatory, as recommended by the French Government Rules, in accordance with Article L1121-1 of the French Public Health guidelines, non-interventional research is not subject to a legal framework. Non-interventional research is defined as any action performed in routine without any additional procedure or unusual diagnostic or monitoring process. Patients were informed that the samples could be used for research purposes. Patients were free to refuse. Samples were used anonymously, with respect for medical confidentiality.

## 3. Results

An STI was found in 16% (95% Confidence Interval: 9.3–22.7) of the patients (19/116), with at least one bacterial strain (CT, NG, or MG) found in one site (the pharynx, rectum, or urine). The prevalence of CT in the pharynx, rectum, and urine was 1% (0–2.8), 8% (3.1–12.9) and 3% (0–6.1), respectively (Table 1). The prevalence of NG in the pharynx, rectum, and urine was 0%, 6% (1.7–10.3) and 1% (0–2.8), respectively. The prevalence of MG was 1% (0–2.8) in rectum and no MG was detected in pharynx, or in urine. One patient was positive in two sites (in urine and in rectum). Among the 99 HIV positive patients, there were eight cases of CT alone (four in rectum, two in urine, one in pharynx, and one in both rectum and urine), two cases of multiple infection CT and NG (both in rectum), one case of multiple infection CT and MG (in rectum), six cases of NG alone (five in rectum, and one in urine), and two cases of syphilis (one of primary grade and one of secondary grade). An STI rate of 17% (9.6–24.4) (17/99) was detected in the HIV positive patients, and an STI rate of 12% (0–27.4) (2/17) was detected in the HIV negative patients (*p* = 0.30). HIV infection was not related to CT, NG, or MG infections in the pharynx, rectum, or urine. 

The prevalence of HR-HPV in the pharynx, rectum and urine was 13% (6.9–19.1), 64% (55.3–72.7) and 6% (1.7–10.3), respectively (Table 1). Seven positive cases were detected in urine; among them, HPV16 was detected in the rectum in five cases, HPV18 was detected in the rectum in two cases (one patient had a multi-infection HPV16 and HPV18), and “other” HR-HPV was detected in one case (result not shown). Fifteen patients were found to be HR-HPV positive in the pharynx; among these, 12 were found HR-HPV positive in rectum, and two in urine (result not shown). HR-HPV was inversely related to HIV positivity only in pharynx (29% (7.4–50.6) of HIV negative cases with HR-HPV *vs.* 10% (4.1–15.9) of HIV positive cases with HR-HPV; *p* (Chi-square test for proportions) = 0.05).

## 4. Discussion

In this cross-sectional study, a high STI rate was found in asymptomatic MSM reporting recent RAI. All of the STI’s were detected, one was detected in one site (MG detected in rectum), one was detected in two sites (NG in rectum and urine), and two were detected in the three sites (CT and HR-HPV). MSM is a particularly susceptible group with high prevalence rates of HR-HPV, HIV infection and other STI’s [5]. Our results are not consistent with the generally known literature: it is generally known that HIV-infected MSM are at increased risk of HR-HPV infection and anal cancer compared with HIV-negative MSM [6,7]. There is a lack of data regarding the HR-HPV infection in MSM HIV negative patients. 

Infection by HR-HPV causes cancer at several anatomical sites; however, the natural history of the infection at non-cervical sites, particularly infection of the oral epithelium, has received little attention from researchers. Kreimer *et al.* recently reported the first natural history study of oral HPV infection [8]. A recent study of oral carriers of HR-HPV infection in men reported that 4.4% (95% CI 3.5–5.6) of these men acquired an oral HR-HPV infection within one year and suggested that within two years over 15% of the men could acquire an oral HR-HPV infection [9]. 

**Table 1:** Anatomic distribution of neisseria gonorrhoeae (GC), chlamydia trachomatis (CT), high risk human papillomavirus (HR-HPV) and mycoplasma genitalium (MG) in the pharynx, rectum and urine, according to HIV infection.

	Pharynx				Rectum				Urine			
	Cohort (*n* = 116)	HIV Negative (*n* = 17)	HIV Positive (*n* = 99)	*p*-Value (HIV Neg. *vs.* HIV Pos.) *	Cohort (*n* = 116)	HIV Negative (*n* = 17)	HIV Positive (*n* = 99)	*p*-Value (HIV Neg *vs.* HIV Pos) *	Cohort (*n* = 116)	HIV Negative (*n* = 17)	HIV Positive (*n* = 99)	*p*-Value (HIV Neg *vs.* HIV Pos) *
CT												
*Neg*	115 (99%)	17	98	0.68	107 (92%)	16	91	0.75	112 (97%)	16	96	0.55
*Pos*	1 (1%)	0	1		9 (8%)	1	8		4 (3%)	1	3	
NG												
*Neg*	116 (100%)	17	99	N/A	109 (94%)	17	92	0.26	115 (99%)	17	98	0.68
*Pos*	0 (0%)	0	0		7 (6%)	0	7		1 (1%)	0	1	
MG												
*Neg*	116 (100%)	17	99	N/A	115 (99%)	17	98	0.68	116 (100%)	17	99	N/A
*Pos*	0 (0%)	0	0		1 (1%)	0	1		0 (0%)	0	0	
HR-HPV												
*Neg*	91 (78%)	12 (71%)	79 (80%)	0.05	23 (20%)	4 (24%)	19 (19%)	0.87	102 (88%)	17 (100%)	85 (86%)	0.24
*Pos*	15 (13%)	5 (29%)	10 (10%)		75 (64%)	12 (71%)	63 (64%)		7 (6%)	0 (0%)	7 (7%)	
*Invalid*	0 (0%)	0 (0%)	0 (0%)	1.00	9 (8%)	1 (6%)	8 (8%)	0.39	1 (1%)	0 (0%)	1 (1%)	0.34
*Not Done*	10 (9%)	0 (0%)	10 (10%)	0.09	9 (8%)	0 (0%)	9 (9%)	0.10	6 (5%)	0 (0%)	6 (6%)	0.15
HR-HPV Types												
16	4 (3%)	1 (6%)	3 (3%)	0.27	3 (2%)	0 (0%)	3 (3%)	0.23	1 (1%)	0 (0%)	1 (1%)	0.34
18	1 (1%)	1 (6%)	0 (0%)	0.007	2 (2%)	0 (0%)	2 (2%)	0.28	1 (1%)	0 (0%)	1 (1%)	0.34
Other	10 (9%)	3 (18%)	7 (7%)	0.07	32 (27%)	5 (28%)	27 (28%)	0.5	4 (3%)	0 (0%)	4 (4%)	0.20
16, 18	0 (0%)	0 (0%)	0 (0%)	1.00	1 (1%)	1 (6%)	0 (0%)	0.007	0 (0%)	0 (0%)	0 (0%)	1.00
16, 18, other	0 (0%)	0 (0%)	0 (0%)	1.00	9 (8%)	2 (12%)	7 (7%)	0.24	0 (0%)	0 (0%)	0 (0%)	1.00
16, other	0 (0%)	0 (0%)	0 (0%)	1.00	19 (16%)	3 (18%)	16 (16%)	0.42	1 (1%)	0 (0%)	1 (1%)	0.34
18, other	0 (0%)	0 (0%)	0 (0%)	1.00	9 (8%)	1 (6%)	8 (8%)	0.39	0 (0%)	0 (0%)	0 (0%)	1.00

* *p*-Values for HR-HPV types comparison between HIV negative and HIV positive was corrected using Bonferroni adjustment. All *p* ≤ 0.001 were considered significant.

Regarding the high prevalence of other STIs, the prevalence of NG was 0% in the pharynx and 6% in the rectum, lower than the prevalence in the pharynx reported by Dudareva-Vizule *et al.* (5.5%); however, the prevalence tends to be higher than the reported prevalence of NG in the rectum (4.6%) [2]. The prevalence of MG found in our study is low in each anatomic locations (0% in pharynx and urine, and 1% in rectum) compared with a prevalence of 5.1% reported by Reinton *et al.* [10]. 

In this study, 16% of the MSM reporting recent RAI were asymptomatic carriers of rectal CT, NG, or MG. A high prevalence of HR-HPV infection was found in the rectum. Among the 99 HIV-infected MSM patients, the STI rate was 17%, with a high risk of transmission over a period of eight months. 

A limitation of this study may be the restricted number of patients due to the short period of inclusion. Nonetheless, our results may be an accurate picture of the actual epidemiology of bacterial STIs in France as reported by La Ruche *et al.* [11].

## 5. Conclusions

According to the high prevalence of asymptomatic STIs found in our MSM population and in other studies, prevention efforts in the form of counseling about the risk of STI needs to be done in the population of MSM [2,10,12]. To stop epidemic STI in MSM patients, systematic screening and treatment should be performed regardless of whether the pathogen detection and the presence of infection *versus* colonization. To inform the prevention efforts for HR-HPV-related oropharyngeal cancers, further investigation of the natural history of oral HR-HPV infection is necessary, focused on the factors associated with oral HR-HPV persistence and clearance. 
